# Spontaneous intracranial hypotension with negative brain MRI findings: a systematic review of diagnostic strategies and clinical outcomes

**DOI:** 10.3389/fneur.2026.1858043

**Published:** 2026-07-09

**Authors:** Marina Romozzi, Giuseppe Garignano, Valid Rastegar, Francesco Onorati, Catello Vollono, Federico Tosto, Masahito Katsuki, Yasuhiko Matsumori, Francesco Signorelli

**Affiliations:** 1 Department of Neuroscience, Università Cattolica del Sacro Cuore, Rome, Italy; 2 Neurology Unit, Dipartimento di Neuroscienze, Organi di Senso e Torace, Fondazione Policlinico Universitario Agostino Gemelli IRCCS, Rome, Italy; 3 Radiology and Neuroradiology Unit, Dipartimento di Diagnostica per Immagini, Radioterapia Oncologica ed Ematologia, Fondazione Policlinico Universitario Agostino Gemelli IRCCS, Rome, Italy; 4 Sidney Kimmel Medical College, Thomas Jefferson University, Philadelphia, PA, United States; 5 Department of Neurosurgery, Fondazione Policlinico Universitario Agostino Gemelli IRCCS, Università Cattolica del Sacro Cuore, Rome, Italy; 6 Department of Neuroscience, “Giovanni Paolo II” Hospital, Lamezia Terme, Catanzaro, Italy; 7 Insight Science Foundation Ireland Research Centre for Data Analytics, School of Human and Health Performance, Dublin City University, Dublin, Ireland; 8 Physical Education and Health Center, Nagaoka University of Technology, Niigata, Japan; 9 Sendai Headache and Neurology Clinic, Sendai, Japan

**Keywords:** brain imaging, fistula, headache, leak, myelography, spontaneous intracranial hypotension

## Abstract

**Background:**

Spontaneous intracranial hypotension (SIH) is a debilitating syndrome typically characterized by orthostatic headache and classic brain MRI findings. However, brain MRI abnormalities are indirect manifestations of cerebrospinal fluid (CSF) volume depletion rather than direct evidence of the leak, and some patients may present with unrevealing or absent typical brain MRI findings, posing a considerable diagnostic challenge.

**Methods:**

A comprehensive search of PubMed/Medline, Web of Science, and Scopus was conducted to perform a systematic review, according to PRISMA guidelines, analyzing the available evidence on MRI-negative SIH (patients without typical SIH findings on their initial brain MRI), including its clinical features, advanced diagnostic strategies, and therapeutic outcomes.

**Results:**

This analysis reveals that, in selected cohorts, brain MRI-negative SIH has been reported in a substantial minority of patients, sharing core clinical symptoms with MRI-positive SIH but often associated with longer symptom duration and higher recurrence rates. While standard qualitative brain MRI without dedicated spinal imaging or dynamic myelographic techniques is often unrevealing, advanced modalities such as CT myelography, MR myelography, and particularly digital subtraction myelography (DSM) may help to identify occult cerebrospinal fluid (CSF) leaks, including CSF-venous fistulas. Treatment with epidural blood patch may provide clinical benefit in selected patients, while targeted surgical repair of demonstrated leaks or CSF–venous fistulas may be associated with high rates of clinical improvement.

**Conclusion:**

In patients with a clinical presentation suggestive of SIH, the absence of typical brain MRI findings should not automatically exclude the diagnosis but should prompt further diagnostic evaluation to improve patients’ outcomes.

**Systematic review registration:**

CRD420251146583. https://www.crd.york.ac.uk/prospero/display_record.php?ID=CRD420251146583

## Introduction

Spontaneous intracranial hypotension (SIH) is a clinical syndrome associated with orthostatic headache. However, the headache pattern can be variable, with patients presenting non-orthostatic and chronic daily headache, particularly later in the disease course (1). It is caused by cerebrospinal fluid (CSF) leakage, historically attributed to reduced intracranial pressure (2). However, several studies suggest that SIH may be more accurately conceptualized as a state of CSF hypovolemia rather than pure hypotension, explaining the cases of SIH with normal intracranial pressure measured at lumbar puncture (3). Gadolinium-enhanced brain MRI is associated with evidence of intracranial pachymeningeal enhancement, brain sagging, venous sinus engorgement, pituitary enlargement, and subdural fluid collections (4, 5). Importantly, the typical cranial MRI abnormalities of SIH should be interpreted as secondary and indirect manifestations of CSF volume depletion rather than as direct evidence of the spinal CSF leak itself (6). Therefore, the absence of typical brain MRI findings should not be interpreted as evidence against SIH in patients with a highly suggestive clinical presentation.

In these cases, advanced imaging techniques, including spinal MRI, MR myelography, CT myelography, and DSM, play an increasingly central role in SIH diagnosis. These modalities allow detection and localization of spinal CSF leaks and CSF–venous fistulas that may not be apparent on standard brain imaging. In selected cohorts, absence of typical brain MRI findings has been reported in a substantial minority of patients; however, these estimates should be interpreted cautiously because definitions of MRI “negativity,” imaging protocols, referral patterns, and diagnostic thresholds varied substantially across studies (2).

This diagnostic scenario, with typical symptoms of SIH associated with unrevealing brain imaging, challenges conventional diagnostic approaches and therapeutic management (7, 8).

Despite the growing recognition of these challenging cases, no previous systematic review has specifically focused on this diagnostic scenario. This diagnostic challenge often leads to delayed recognition, inappropriate management, and variable outcomes.

Our systematic review aims to analyze the available evidence on patients with SIH, presenting with an initial brain MRI without typical findings, comparing their distinctive features with MRI-positive cases, discussing the role of advanced imaging in diagnosis, and evaluating therapeutic outcomes and implications for clinical management.

## Methods

### Search strategy and selection

The systematic review was performed according to Preferred Reporting Items for Systematic Reviews and Meta-Analyses (PRISMA) 2020 guidelines (9) to investigate the diagnostic work-up of patients with SIH and CSF hypotension presenting without typical MRI findings. A comprehensive literature search of PubMed/Medline, Web of Science, and Scopus was performed on 3rd September 2025 for Human studies published in English. This systematic review was registered and accepted in PROSPERO database with the following ID: CRD420251146583.

**Figure 1:**
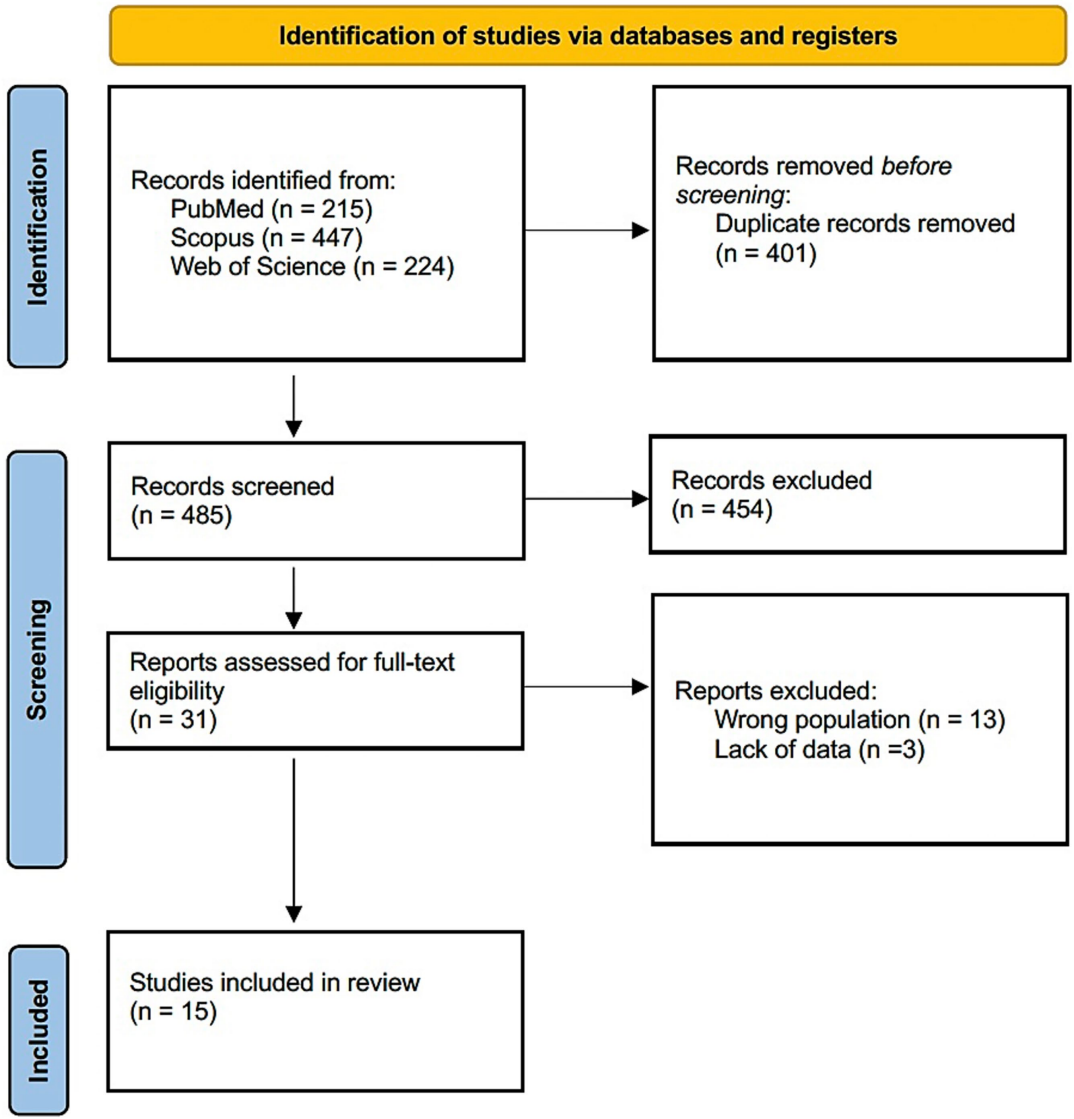
PRISMA flowchart depicting the study selection and screening process.

After searching the three databases, all results were collected and uploaded to Rayyan (10), where duplicates were also automatically removed. Two authors (F.O., F.T.) independently screened the titles and abstracts. Three reviewers (F.O., V.R., F.T.) independently screened the full texts of selected articles, and a senior author (M.R.) resolved discrepancies. The PRISMA flowchart (Figure 1) depicts the selection and screening process.

### Eligibility criteria

To meet the inclusion criteria, articles had to: (1) Report or include patients with SIH or CSF hypotension presenting without typical brain MRI findings; (2) be English peer-reviewed articles; and (3) be original studies reporting diagnostic work-up, treatment, and outcome. Exclusion criteria included: (1) gray literature (i.e., conference abstracts and dissertations); (2) review articles; (3) animal-based studies; and (4) studies with no available English translation. The terms “SIH” and “CSF hypotension” were both included in the search strategy to capture studies in which these terms were historically used interchangeably (Supplementary materials). However, they were not considered strictly synonymous. CSF hypotension refers primarily to low CSF pressure, whereas SIH is a broader clinical syndrome that may occur with normal opening pressure and may be better conceptualized in some cases as CSF volume depletion or CSF hypovolemia. Therefore, we checked all included cases and only patients fulfilling a clinical diagnosis of SIH were considered, regardless of whether the original studies used the term “CSF hypotension.” For the purposes of this review, “spontaneous” was used to indicate cases in which no recognized iatrogenic, traumatic, post-procedural, or secondary cause of CSF leakage was reported in the original study. When available, we extracted whether such causes had been explicitly excluded.

In this review, we use the term “MRI-negative” to indicate patients in whom the initial brain MRI evaluation did not show typical SIH-related brain signs. This category includes heterogeneous situations, such as non-contrast or contrast-enhanced MRI interpreted as unrevealing, qualitatively normal scans later recognized as subtly abnormal, and cases in which typical cranial findings appeared only on follow-up imaging. Therefore, “MRI-negative” represents a real-world diagnostic scenario in which standard brain imaging fails to provide typical supportive evidence of SIH.

### Data extraction

Data extraction was performed on each included study using its respective text, tables, and figures. The Population, Intervention, Comparison, and Outcome (PICO) framework was utilized. The study population (P) was defined to include patients with suspected SIH who presented with a negative brain MRI. The intervention (I) involved advanced diagnostic modalities such as CT myelography, DSM, MR myelography with intrathecal contrast, and radionuclide cisternography. The comparison group (C) included patients undergoing standard initial diagnostic assessments, such as conventional MRI alone or clinical evaluation without advanced imaging. The primary outcome (O) to be extracted from the studies included was the confirmation of SIH diagnosis, including the localization of CSF leak (spine or brain), diagnostic accuracy of the diagnostic modality, time to diagnosis, and impact on the management of SIH. Additional data extracted included demographic information of patients, presenting symptoms, treatment, and outcome. We also extracted the level of diagnostic support for SIH and classified cases, when possible, as: confirmed SIH, when a spinal CSF leak, extradural CSF collection, or CSF–venous fistula was objectively demonstrated; probable SIH, when diagnosis was supported by typical clinical features together with low opening pressure, indirect imaging findings, follow-up evolution, or treatment response; and clinically suspected SIH, when diagnosis was based mainly on orthostatic headache despite negative brain and spinal imaging.

Data extraction was conducted by two investigators (F.T. and F.O.). Subsequently, a senior author (M.R.) independently reviewed and double-checked the extracted data from all articles. Discrepancies between the investigators were resolved through discussion and consensus.

### Risk of bias

We applied the ROBINS-I V2 (11) (Risk of Bias in Non-randomized Studies of Interventions, Version 2) tool, along with the ROBINS application^1^, for bias evaluation through visual representation. In addition, study quality was assessed using the GRADE (Grading of Recommendations Assessment, Development and Evaluation) (12) approach via the GRADEpro website^2^. Any discrepancy was solved through discussion and consensus.

### Data synthesis

A qualitative, narrative synthesis of the extracted data was performed due to the heterogeneity of the included studies. A quantitative meta-analysis was not considered appropriate given the variability in study designs (e.g., case series, observational studies), patient populations, diagnostic modalities employed, and the inconsistent reporting of outcome measures across the studies.

The findings are presented thematically to address the primary objectives of this review. The synthesis focuses on summarizing: (1) the characteristics of the included studies; (2) the clinical features of patients with MRI-negative SIH; (3) the diagnostic approaches used in the absence of typical MRI findings; (4) the treatments administered and their corresponding outcomes; and (5) data related to prognosis and recurrence rates.

## Results

### Study selection

The initial literature search yielded a total of 886 results across three databases (PubMed, Scopus, and Web of Science). After automatic removal of duplicates using Rayyan, 485 studies remained and were screened for title and abstract. Title and abstract screening resulted in the exclusion of 454 articles. The full text of the remaining 31 studies was assessed for eligibility, of which 16 were excluded (13 = wrong population, 3 = lack of data). Ultimately, a total of 15 studies were included in the final qualitative synthesis. The detailed flowchart summarizing this process is provided in Figure 1.

### Characteristics of included studies

At the title and abstract screening phases, records were primarily excluded because they report post-procedural or traumatic CSF leaks, focused exclusively on SIH with positive imaging, were narrative reviews or technical reports, or lacked sufficient clinical or imaging detail to assess MRI-negative presentations. Moreover, evaluation of quantitative or structured brain MRI scoring systems could not be uniformly applied, as most included studies did not include this evaluation or did not report the necessary imaging parameters.

The 15 included studies were published between 1999 and 2025 and comprised a total of 529 patients, of which 266 were classified in the original studies as MRI-negative, meaning absent, normal, negative, or initially unrevealing typical brain MRI findings, while the clinical diagnosis of SIH was supported by symptoms, additional imaging, treatment response, or follow-up. The number of patients in each study ranged from 1 to 136. Diagnostic certainty varied substantially across the included studies. Some studies included patients with objectively demonstrated spinal CSF leakage, extradural CSF collections, or CSF–venous fistulas, whereas others included patients with low opening pressure, supportive but indirect imaging findings, follow-up evolution, or clinical response to epidural blood patch. Detailed characteristics of each included study are summarized in Table 1.

**Table 1:** Clinical, imaging, treatment, and outcome characteristics.

Author/year	Mean age (years)	Number of patients	Brain MRI protocol and MRI-negative definition	Presenting symptoms	Brain MRI	Brain MRI-negative cases	Spinal/myelographic/radionuclide work-up	Objective leak/CVF evidence	Treatment	Clinical/imaging outcome
Akbar et al. (2012) (13)	51	41	Gd + brain MRI in all patients before GdM Brain MR imaging was graded as “equivocal” if PE without “brain sag” or “brain sag” without PE was noted. Brain MR imaging was graded as negative if neither PE nor sagging brain was present.	Orthostatic headache	In CTM– group: 14 with classic signs (PE + BS), 3 equivocal (one sign only), 6 negative. Among 5 GdM-positive: 2 classic, 1 equivocal, 2 negative	6/24 CTM– had negative MRI; 1/6 leak localized by GdM	Extradural fluid in 8/36; 2/21 CTM–. 1/5 GdM-positive also had extradural fluid	GdM localized leaks in 5/24 CTM– (thoracic T1–T4, lumbar L1–L2); recurrent leaks confirmed in 12/17 CTM+	Conservative, EBP, fibrin glue, surgery	3/4 targeted EBP improved; fibrin glue failed; recurrence resolved with EBP;
Lee et al. (2018) (14)	34	1	Brain MRI and MRA contrast use not clearly specified. Negative in absence of typical findings	Orthostatic headache, nausea, dizziness, eye pain	Brain MRI and MRA normal	Patient had no abnormal findings	No spinal MRI; radionuclide cisternography done: multiple leaks (lumbar + upper thoracic)	Lumbar L3–5, thoracic T3–4	EBP	Symptoms resolved
Choi et al. (2023) (8)	40	47	Gd + brain MRI in all patients. Negative in absence of typical findings.	Orthostatic headache	MRI with gadolinium normal in all	47/121 normal findings	21 underwent EBP: CTM 5, MR myelography 16: all negative	Not detected	EBP	At discharge 14/21 improved ≥50%; at 3 months 19/21 improved, 11/21 complete remission
Mokri et al. (1999) (16)	37.2	6	Gd + brain MRI in all patients. Negative in absence of typical findings.	All with orthostatic headache; some with nausea, neck pain, tinnitus, muffled hearing	5/6 positive at baseline (tonsillar descent/BS or PE)	1/6 — one patient had initially normal head MRI; PE appeared 2 weeks later	5/6 reported —Extra-arachnoid/extradural CSF collections; diverticula at T7 and T12 in one; spine MRI was initially normal in patient with initially normal brain MRI	C6 root sleeve (patient 4), T9 root sleeve (patient 2), T8 thoracic (patient 6); others not precisely localized	EBP, laminectomy, fibrin glue, conservative	Improvement or resolution in 5/6, 1/6 no improvement.
Schievink et al. (2005) (18)	41	33	Gd + brain MRI in all patients. Negative in absence of typical findings.	31 positional headache, 1 non-positional headache, 1 neck pain	26/33 abnormal: PE (24), BS (17), subdural collections (14).	7/33 normal	CTM in all: all had leaks; multiple in most cases	Cervical (16), thoracic (15), lumbar (2); all normal brain MRI– cases had multiple leaks	Conservative (2), EBP (24), fibrin glue (6), surgery (13)	Good outcome in 25/26 with abnormal MRI vs. 1/7 (14%) with normal Brain MRI
Schievink et al. (2013) (19)	14.3	24	Gd + brain MRI in all patients. Negative in absence of typical findings.	23/24 orthostatic headache 1 non positional headache; common: nausea/vomiting (67%), neck pain/stiffness, dizziness, aural symptoms (33%); some visual symptoms, ataxia	Abnormal in 19/24: BS, PE, subdural hematomas, pituitary hyperemia, VSE.	5/24 negative brain MRI	Spinal MRI/MR myelography in all patients, CTM 20, RNC 5, CSF leak in 12; meningeal diverticula/dural ectasia in 10; normal in 2	Thoracic predominant, often ventral leaks; meningeal diverticula common	EBP, fibrin glue injections, surgeries.	Good outcome in 22/24 poor in 2. 5 developed rebound intracranial hypertension; 2 had recurrences years later
Schievink et al. (2021) (17)	46	60	Gd + brain MRI in all patients. Negative in absence of typical findings.	Orthostatic headache	Brain MRI normal in all	60/60 negative (by design)	Conventional spine MRI/CTM before DSM; No extradural CSF; DSM identified CSF–venous fistula in 6/60	All fistulas were thoracic; 6/31 with meningeal diverticula	Most had prior EBP; CVF patients had surgical ligation	5/6 complete resolution, 1 partial recurrence
Schievink et al. (2024) (20)	47.5	93	Gd + brain MRI in all patients. Negative in absence of typical findings.	Orthostatic headache	Brain MRI normal in all	93/93 negative	Conventional MRI/myelography before DSM; No extradural CSF; DSM identified CVF in 15/93 (16%)	All thoracic CVFs (9 right, 6 left)	All 15 underwent surgical ligation	Post-op: ONSD ↑ 4.0 → 5.3 mm, SAS ↑ 0.5 → 1.2 mm; 80% complete/near-complete resolution, 13% no improvement
Shah et al. (2013) (21)	45.3	29	Gd + brain MRI in all patients. Some cases were defined as “qualitatively” negative but showed abnormal quantitative metrics	Orthostatic headache; nausea, vomiting, neck pain, tinnitus, vertigo, visual/hearing disturbance	Qualitative: PE 17/29 (59%), VSE 14/29 (48%), subdural collections 9/29 (31%), tonsillar herniation 14/29 (44%). Quantitative: mean pontomesencephalic angle 41.2° (controls 65°), mamillopontine distance 4.4 mm (controls 7.0 mm); cut-offs ≤50° and ≤5.5 mm diagnostic	1/29 had qualitatively negative MRI, but showed abnormal quantitative metrics	Not reported	Not always identified; some postsurgical/shunt-related, others spontaneous	EBP, shunt adjustment in shunt related case	10/29 improved after ≥1 EBP; 6/28 shunt-related improved after shunt adjustment
Schoffer et al. (2002) (7)	42.3	4	Gd + brain MRI in all patients. Negative in absence of typical findings.	Orthostatic headache (all); some with nausea, vomiting, ataxia, facial paresthesia	3/4 had classic MRI changes (diffuse PE, subdural/extradural collections, tonsillar descent).	1/4 had normal MRI despite typical symptoms and low CSF pressure	Spine MRI in patients with positive MRI showed subdural/extradural collections; in MRI negative case: no abnormalities Subdural/extradural collections in 3 cases; none in the MRI-negative case	Not anatomically localized	Conservative (IV fluids, bed rest, antiemetics)	All recovered with conservative management; complete resolution of symptoms within weeks–months, including MRI abnormalities
Lee et al. (2022) (15)	48	136	Gd + brain MRI in all patients. Negative in absence of typical findings.	Orthostatic headache (88%), nausea/vomiting (67%), neck pain (42%), dizziness (33%), tinnitus (29%)	128/136 brain MRIs: VSE (65%), pituitary enlargement (63%), PE (57%), sagging (42%), subdural collection (21%).	24/128 normal brain MRI	MR myelography in all: 105/120 (88%) confirmed leaks localized; epidural fluid in 90/120 (75%)	Thoracic 64%, lumbar 52% (iatrogenic), thoracolumbar 29%, cervical 28%, craniocervical 21%	EBP in 116/136;	90% improved overall; MRI negative subgroup: 18/19 improved
Udommongkol et al. (2005) (22)	54	1	Gd + brain MRI in all patients. Negative in absence of typical findings.	Orthostatic headache, nausea, dizziness	Brain MRI with gadolinium normal	Brain MRI with gadolinium normal	No spine MRI; radionuclide cisternography done; 3 lumbar leaks + early bladder activity	Multiple lumbar	EBP	Complete remission in 2 days, no relapse at 3 years
Wiesemann et al. (2006) (23)	50	10	Gd + brain MRI in all patients. Negative in absence of typical findings.	6/10 orthostatic headache; others: tinnitus, gait ataxia	7/10 abnormal: PE (7), subdural collection (2), BS (4)	3/10 negative MRI; all confirmed SIH by cisternography	Spine MRI in 6/10; 3 showed extra-arachnoid fluid/root sleeve dilatation; RNC abnormal in all; 6 focal leaks, others abnormal dynamics	Leaks at cervicothoracic, thoracolumbar, lumbar	Conservative (3), neurosurgical drainage (2), EBP (4), dialysis modification (1)	Most recovered; EBP effective in 3, surgery in 2, 1 improved post-transplant; MRI negativity did not predict poor outcome
Yoon et al. (2025) (24)	44	42	Gd + brain MRI in all patients. Negative in absence of typical findings.	81% orthostatic headache	Group 1 (27) brain MRI positive: VSE 92.6%, PE 66.7%, BS 7.4%, subdural 37%. Group 2 (15) brain MRI negative:	15/42 negative	Standardized CE-CTL spine MRI in all; SLEC 97.6%, DE 97.6%, EVE 95.2%, C1–2 sign 73.8%, high SI cervical 66.7%. MRI– group: more partial/posterior SLEC, thoracic predominance	Leak site not anatomically specified; thoracic predominance; CVF suspected in MRI– cases	95% received EBP; 2 conservative	59.5% improved; 40.5% recurrence/persistence. MRI negative group had longer headache duration and higher recurrence
Zhang et al. (2025) (25)	63	2	Brain MRI and MRA in case 1. Gd + brain MRI in case 2. Negative in absence of typical findings.	Orthostatic headache progressing to encephalopathy and coma; dysarthria, imbalance, dysphagia, urinary incontinence	Case 2: PE, VSE, BS, subdural collections (Bern 9/9)	Case 1: Initial and subsequent MRI with Gd contrast interpreted as negative for SIH; retrospective review revealed pituitary hyperemia, VSE, brainstem distortion (Bern 6/9)	Case 1: thin ventral epidural collection C6–T9; Case 2: ventral epidural collection T8–T10 with smaller lumbar collections	Case 1: ventral dural tear T3–T4 with disk-osteophyte. Case 2: ventral dural tear T12–L1 with disk-osteophyte	Trendelenburg positioning reversed coma in both; both underwent surgical repair (laminectomy + dural repair)	Complete neurologic recovery in both; no deficits at 1 month

BS, brain sagging; CE-CTL MRI, contrast-enhanced cervical-thoracic-lumbar magnetic resonance imaging; CSF, cerebrospinal fluid; CTM, CT myelography; CVF, CSF–venous fistula; DE, dural enhancement; DSM, digital subtraction myelography; EBP, epidural blood patch; EVE, engorged venous plexus; Gd+, Gadolinium-enhanced; GdM, gadolinium-enhanced myelography (intrathecal); MRA, magnetic resonance angiography; MRI, magnetic resonance imaging; ONSD, optic nerve sheath diameter; PE, pachymeningeal enhancement; RNC, radionuclide cisternography; SAS, subarachnoid space (perioptic); SI, signal intensity; SIH, spontaneous intracranial hypotension; SLEC, spinal longitudinal extradural CSF collection; VSE, venous sinus engorgement.

Across the included studies, we grouped MRI-negative cases into four categories: (1) patients with initially unrevealing non-contrast brain MRI; (2) patients with no typical findings on qualitative brain MRI assessment despite clinically suspected or confirmed SIH; (3) patients with subtle or retrospectively recognized cranial abnormalities; and (4) patients with initially negative brain MRI who developed typical findings on follow-up imaging. Because these categories were inconsistently distinguished in the original studies, we treated MRI negativity as a diagnostic scenario rather than as a homogeneous disease subgroup. Moreover, information on structured imaging assessment and longitudinal validation was inconsistent across studies. Some studies used more standardized or quantitative approaches, including predefined brain MRI categories, MR myelography detection rates, optic nerve sheath measurements, or standardized whole-spine MRI protocols. However, post-treatment imaging normalization and structured pre/post-treatment comparisons were available only in selected studies. Therefore, the extent to which subtle cranial or spinal findings represented dynamic SIH-related abnormalities rather than nonspecific or anatomical variants could not be systematically assessed across the whole literature.

### Clinical features of MRI-negative spontaneous intracranial hypotension

The most universally reported presenting symptom among patients with MRI-negative SIH was orthostatic headache, which was present in most patients across all studies (7, 8, 13–25). Other commonly reported symptoms included nausea and vomiting (7, 14–16, 19, 21, 22), neck pain or stiffness (15, 16, 18, 19, 21), dizziness (14, 15, 19, 22), tinnitus (15, 16, 21, 23), and visual symptoms (19, 21). In rarer cases, patients presented with severe neurological signs such as ataxia (7, 19, 23).

Based on the information available in the original studies, a total of 529 patients with suspected SIH were evaluated. 266 patients were considered as MRI-negative cases and were categorized in four categories: (1) 1 case with initially unrevealing routine brain MRI and unclear contrast information (14); (2) 262 cases with no typical SIH-related findings on brain MRI despite clinical, spinal imaging, pressure, treatment-response, or follow-up support (7, 8, 13, 15, 17–20, 22–24); (3) 2 cases with subtle, retrospectively recognized, or quantitatively abnormal cranial findings despite initially qualitative-negative interpretation (21, 25); and (4) 1 case with initially negative brain MRI that became positive on follow-up imaging (16). Because the studies inconsistently reported MRI protocol, contrast use, timing of imaging, rereading, and follow-up, these categories should be considered descriptive rather than mutually exclusive subgroups.

Notably, we included studies where the initial MRI was described as unrevealing during an early phase. Therefore, when referring to MRI-negative cases, we specifically considered the first MRI examination, which is often performed without contrast. Although subsequent imaging may later become positive or be repeated with contrast enhancement, we believe it is important to describe the initial imaging assessment, as this is the examination most commonly used in routine clinical practice. For instance, Mokri et al. (16) reported a case in which the initial brain MRI(use of gadolinium not specified) was normal. In this case, baseline post-contrast evaluation was inferred from the initial report of normal meningeal findings and from the subsequent development of pachymeningeal enhancement on follow-up brain MRI 2 weeks later. Similarly, Zhang et al. (25) described a case where the patient presented only orthostatic headache and initial brain MRI was deemed unrevealing, but a later observation resulted in the identification of subtle signs of SIH, such as pituitary hyperemia and venous sinus engorgement. This report should not be interpreted as evidence of severe SIH with persistently normal brain MRI, but rather as an example of real-world scenarios, where an initially presumptive unrevealing standard brain imaging was followed by the emergence of intracranial signs upon severe clinical deterioration. Moreover, Shah et al. highlighted the interpretive limitations of routine qualitative brain MRI assessment, showing that cases initially considered MRI-negative may be reclassified as abnormal when quantitative intracranial measurements are applied (21).

### Diagnostic approaches in the absence of MRI findings

Information on the interval between symptom onset and brain MRI acquisition was inconsistently reported across studies.

In patients with MRI-negative presentation, various diagnostic modalities were employed to confirm the diagnosis of SIH and localize the CSF leak. The most frequently utilized techniques were CT myelography (CTM) (8, 13, 17–19), MR myelography (8, 15), and radionuclide cisternography (RNC) (14, 22), which were variably useful in identifying extradural fluid collections, sites of CSF leakage along the spinal axis, and meningeal diverticula, a finding not considered direct evidence of active CSF leakage (Table 1).

In more recent studies, DSM was utilized to identify CSF-venous fistulas (CVF), particularly when conventional spine MRI and myelography were normal (17, 20). Schievink et al. (17, 20) identified thoracic CVF in 10% (6/60) and 16% (15/93) of cases undergoing DSM after negative conventional spinal imaging, respectively. In contrast, Choi et al. (8) reported that even advanced diagnostic modalities such as CTM and MR myelography were negative in their cohort of 21 patients. Hence, the diagnosis of “probable SIH” was established based on clinical presence of orthostatic headache without any imaging abnormality (Table 1).

### Treatment and outcomes

The management strategies used for MRI-negative SIH were varied and included conservative measures (bed rest, hydration) (7, 13, 16, 18, 23, 24), epidural blood patches (EBP) (8, 13–16, 18, 19, 21–24), fibrin glue injections (13, 16, 18, 19), and surgical repair (13, 17–20, 23, 25). EBP was the most common intervention performed, reported as treatment in 11 studies.

Different outcomes were reported across the studies, with 2 studies reporting significant or complete symptom resolution in all patients following targeted or non-targeted EBP (14, 15). Notably, Schievink et al. (18) reported a significantly poorer outcome in patients with normal brain MRI (improvement in 14% of cases) compared to those with abnormal MRI findings (improvement in 97% of cases). Surgical ligation of CVF patients had a favourable outcome, with most patients showing complete or near-complete resolution (17, 20). A summary of comparing the findings between MRI-positive and MRI- negative SIH can be found in Table 2.

**Table 2:** CSF, cerebrospinal fluid; CTM, CT myelography; DSM, digital subtraction myelography; EBP, epidural blood patch; MRI, magnetic resonance imaging; SIH, spontaneous intracranial hypotension; Clinical and imaging features of MRI-positive vs. MRI-negative SIH cases.

Feature	MRI-positive SIH	MRI-negative SIH
Prevalence	Majority of SIH cases	Approximately 20%–35% of SIH patients
Headache type	Typically orthostatic	Typically orthostatic
Onset	Acute or subacute	Acute or subacute, but diagnosed late
Associated symptoms	Nausea, neck stiffness, hearing loss, diplopia, tinnitus	Similar but often less pronounced
Severe neurological signs	Rare (ataxia, coma)	Reported in isolated cases (ataxia, encephalopathy, coma)
Spinal imaging	Often shows extradural CSF collections, dural tears, or meningeal diverticula	Frequently normal on conventional imaging; may reveal CSF–venous fistula on DSM
Type of CSF leak	Dural tear or leaking meningeal diverticulum	CSF–venous fistula (no extradural CSF collection)
Opening pressure (lumbar puncture)	Low (<60 mmH₂O)	Normal or low-normal (‘normotensive SIH’)
Diagnostic challenges	Diagnosis usually straightforward with typical MRI findings	Diagnosis frequently delayed; often requires CTM, MR myelography, or DSM
Most useful diagnostic modality	Standard MRI and spinal imaging	DSM, MR myelography with intrathecal contrast
Treatment	Conservative measures and non-targeted EBP often effective	Frequently require targeted EBP or surgical ligation of fistula
Response to EBP	Good to excellent	Good; variable if CSF-venous fistula untreated
Surgical outcomes	Usually not required	Excellent results after targeted surgical repair of CSF–venous fistula
Symptom duration before diagnosis	Shorter, due to typical MRI findings	Longer; delayed recognition common
Recurrence rate	Moderate (~10%–15%)	Higher recurrence and symptom persistence reported
Prognosis	Favorable with early recognition and treatment	Favorable if source localized and treated; poorer if diagnosis delayed

### Prognosis and recurrence rates

The long-term prognosis for patients with MRI-negative SIH appears generally favourable, although recurrences can occur. Yoon et al. noted that the MRI-negative group in their study had a higher rate of recurrence and symptom persistence in addition to longer headache duration compared to the MRI-positive group (24). Akbar et al. (13) also reported recurrence, with symptoms improving after repeated EBP.

### Risk of bias

**Figure 2:**
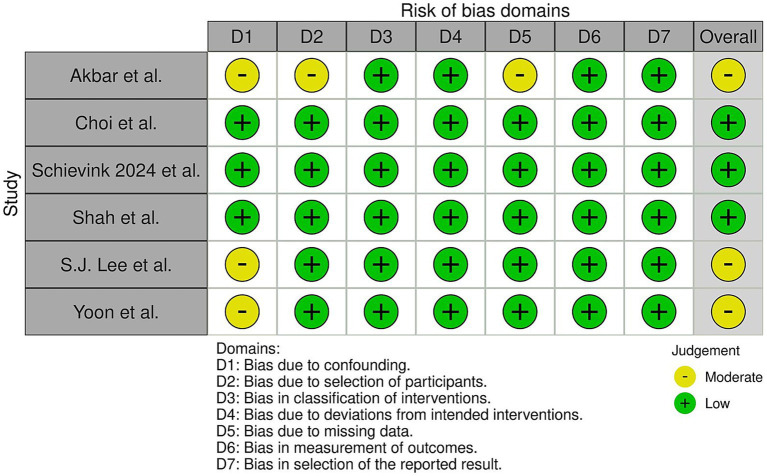
Risk of bias assessment using the ROBINS-I-V2 tool.

Risk of bias was assessed using the ROBINS-I-V2 (11) tool for six studies (8, 13, 15, 20, 21, 24), in which an intervention and a control group could be identified. Overall, the risk of bias was judged as moderate to low, with the main concerns related to potential confounding, due to non-randomized designs and lack of adjustment for baseline differences, such as the severity of the clinical presentation, since patients with a less evident clinical picture of SIH and negative MRI could have been less likely to undergo advanced imaging or to have it delayed, possibly influencing the detection outcome. The other domains were generally considered low risk (Figure 2).

The remaining studies were included in the quality appraisal, which was performed using the GRADE (12) tool. The primary concern was indirectness, as most studies were designed to answer research questions unrelated to those of our review, leaving only a fraction of their populations relevant to the scope of this article. Additionally, the absence of a proper diagnostic gold standard further limited the ability to calculate key parameters, such as sensitivity and specificity, reducing the overall certainty of the evidence.

## Discussion

From our review of the literature, a key concept that emerges is not that SIH occurs paradoxically despite normal brain MRI, but that standard cranial MRI can be unrevealing and may have limited sensitivity as an indirect marker of CSF volume depletion. Typical brain MRI findings are secondary intracranial manifestations of altered CSF volume and craniospinal compliance (26), whereas the primary diagnostic target remains the identification of a spinal CSF leak, CSF–venous fistula, or other CSF volume disorder (2). Therefore, in patients with typical clinical features, an unrevealing brain MRI should be interpreted as the absence of evident secondary cranial signs rather than as exclusion of SIH. An additional source of heterogeneity concerns diagnostic certainty. MRI-negative SIH in the included literature did not represent a uniform diagnostic category. Some patients had objective evidence of spinal CSF leakage or CSF–venous fistula, whereas others were diagnosed based on low opening pressure, supportive indirect imaging findings, follow-up evolution, or treatment response (7, 8, 21, 24). This distinction is clinically important because outcomes and recurrence rates may differ between anatomically confirmed leak-positive cases and clinically suspected imaging-negative cases without demonstrable leakage (24). Treatment response to EBP may support the diagnosis in selected patients, but it should not be considered equivalent to direct anatomical confirmation, because spontaneous fluctuation, conservative measures, placebo effects, or nonspecific procedural effects may also influence symptoms (27).

In addition, an important issue concerns the diagnostic interpretation of subtle cranial and spinal imaging findings. Several SIH-related imaging signs are relative rather than absolute abnormalities, and their diagnostic relevance may depend on the clinical context, imaging protocol, timing from symptom onset, scanner quality, and reader expertise (28). Typical brain MRI findings may support the diagnosis when concordant with the clinical picture, but they may be difficult to interpret in isolation. This is particularly relevant because pre-symptom imaging is rarely available, making it difficult to determine whether subtle findings represent true SIH-related changes or anatomical or nonspecific variants.

Akbar et al. reported 6 out of 24 CT myelography-negative patients with a normal brain MRI, only one of whom had the leak localized by intrathecal gadolinium MR myelography (13). Schievink et al. (19) observed that 5 of 24 children and adolescents had no abnormal cranial findings despite typical orthostatic headache and, in some, documented spinal leaks. In the large cohort of Lee et al. (15), 24 of 128 patients (19%) were brain MRI–negative, but two-thirds of them showed leaks on spinal imaging. Yoon et al. (24) found 15 of 42 patients investigated (35.7%) with normal brain MRI. By design, all patients in the series of Schievink et al. (2021 and 2024) had negative brain MRI (17, 20). In addition, Mokri et al. (16) showed that the absence of pachymeningeal enhancement could be transient, with patients showing normal scans at baseline and enhancement only at follow-up imaging performed weeks later, while Schoffer et al. (7) described one patient with normal brain MRI but low opening pressure on lumbar puncture, recovering with conservative treatment.

Taken together, these data suggest that a substantial minority of patients with clinically suspected or confirmed SIH may lack typical signs on initial brain MRI. However, the true frequency remains uncertain because of heterogeneous imaging protocols, diagnostic thresholds, and definitions of MRI negativity. These observations support a dynamic model of SIH imaging, in which brain MRI findings may be negative at initial imaging evaluation of the brain and evolve over time.

Clinically, the presentation of MRI-negative patients overlaps with that of MRI-positive cases. Orthostatic headache remains the main symptom in both MRI-positive and MRI-negative patients. Moreover, it is also frequently accompanied by nausea, neck pain, tinnitus, or vestibular disturbances. Notably, in the work of Yoon et al., it emerged that MRI-negative patients tended to have longer symptom duration and a higher recurrence rate (24). Interestingly, Choi et al. reported cases with normal brain and spine MRI that improved after empiric EBP, suggesting that empiric EBP may be considered in carefully selected high-probability cases after exclusion of alternative diagnoses, although response should not be interpreted as equivalent to anatomical confirmation of a CSF leak (8).

Initial MRI-negative patients in real-world scenarios can also be detected in cases that can evolve in a severe clinical picture. Zhang et al. described two patients presenting with coma due to SIH caused by ventral dural tears, one of whom had initial normal brain imaging before a more structured re-examination revealed subtle abnormalities. Of note, the two patients demonstrated remarkable recovery after placement in Trendelenburg position (25). This initial misinterpretation reveals the complexity of standard brain MRI evaluation in diagnosis of SIH and reflects the importance of a comprehensive imaging evaluation in cases with symptoms compatible with SIH, which may help prevent progression toward severe neurological deterioration.

Available data suggest that outcomes may differ between patients with and without typical brain MRI findings, although this comparison is limited by heterogeneous case definitions, different treatment strategies, and variable diagnostic certainty. Schievink et al. (18) demonstrated that patients with abnormal brain MRI had good outcomes in 97% of cases, while only 14% of those with normal MRI improved, all of whom harboured multiple spinal leaks. This suggests that in some patients the absence of cranial abnormalities, when a leak is present, may reflect a more complex or multifocal leak pattern that is harder to treat. By contrast, in the paediatric population studied by Schievink et al. (19) and in the large series of Lee et al. (15), MRI-negative patients who underwent targeted treatment based on spinal imaging often had good outcomes. These data suggest that prognosis relies more on localization and closure of the leak rather than the presence of brain MRI typical findings.

Several aspects may explain a negative brain MRI in SIH. First, temporal factor plays an important role: Mokri et al. (16) documented patients in whom enhancement appeared only on repeat imaging, while in others, enhancement disappeared despite persistent leak. Interpretive limitations are another factor: Shah et al. showed that two patients with qualitatively normal MRI, i.e., routine visual evaluation of brain MRI without the use of structured scoring systems or quantitative angle and distance measurements, were found abnormal using quantitative measurements of pontomesencephalic angle and mamillopontine distance (21), and Zhang et al. (25) highlighted how structured scoring can upgrade scans initially labelled as normal. Notably, abnormal findings on spine imaging can be found even in the absence of brain involvement at MRI. Yoon et al. (24) reported that MRI-negative patients can present with partial or posterior spinal longitudinal extradural CSF collections, with thoracic prevalence. It is worth mentioning the presence of cases characterized by occult spinal sources such as CSF-venous fistulas. Schievink et al. (2021 and 2024) reported a series of cases that showed how normal conventional MRI can be present in patients with thoracic fistulas, a finding usually associated with clear intracranial hypotension signs on routine qualitative assessment, detectable with targeted techniques such as CT myelography or DSM, and treatable with surgical ligation (17, 20).

Longitudinal imaging may help address this uncertainty. In Lee et al. (15), follow-up brain MRI supported the dynamic nature of cranial MRI signs in SIH, with changes in typical findings over time. Similarly, Schievink et al. (20) showed that optic nerve sheath diameter and perioptic subarachnoid space measurements increased after surgical ligation of CSF–venous fistulas, providing quantitative evidence that some subtle perioptic markers may normalize after successful treatment. Akbar et al. also highlighted the relevance of technique and timing, as delayed intrathecal gadolinium MR myelography localized leaks in selected patients after negative CT myelography, including one case with negative brain MRI, spine MRI, and CT myelography (13). However, such longitudinal, delayed, or post-treatment imaging validation was not systematically available across the included studies, limiting the ability to determine whether subtle imaging findings represented true SIH-related dynamic changes or nonspecific variants.

Advanced imaging techniques, therefore, play a central role in the diagnostic pathway. Intrathecal gadolinium MR myelography, as in Akbar et al., localized leaks after negative CT myelography in approximately one-fifth of cases (13). MR myelography without contrast, as reported by S. J. Lee et al., showed high sensitivity in detecting leaks even in the case of normal brain MRI (15). DSM is regarded as one of the most sensitive techniques for identifying CSF-venous fistulas, especially in the presence of meningeal diverticula, and is recommended in case of negative conventional studies (17, 20). Standardized, contrast-enhanced, whole-spine MRI protocols also increase diagnostic yield, as demonstrated by Yoon et al., who were able to recognize patients with SIH after detecting subtle spinal findings (24).

Looking at treatment options, an MRI-negative status does not exclude response to standard treatment. In the series of Choi et al., empiric EBP produced substantial and sustained benefit in most patients despite negative imaging (8). Moreover, when a venous fistula or ventral dural tear is identified, surgical repair usually leads to good outcomes, as shown by Schievink et al. (2021, 2024) (17, 20) and Zhang et al. (25). On the other hand, the poor results in the MRI-negative subgroup of Schievink et al. (18) and the lack of consistent benefit in patients without demonstrable leaks reported by Leep Hunderfund and Mokri (29) underline the risks of empiric procedures without anatomical confirmation. Case reports demonstrated that radionuclide cisternography or repeat advanced imaging can reveal leaks in MRI-negative presentations, with variable but sometimes curative outcomes (14, 22, 23).

### Limitations and future directions

This review is limited by the heterogeneity of included studies, frequent retrospective designs, small sample sizes, different and unspecified timing in imaging evaluation, and the lack of systematic use of structured MRI scoring systems. Additionally, defining MRI negativity based on the initial non-contrast MRI may lead to misclassification, as some cases may become positive on follow-up or with contrast.

Future studies should use prospective multicenter designs with standardized brain and spine MRI protocols, structured scoring systems, quantitative measurements, expert rereading, and longitudinal post-treatment imaging. Such approaches are needed to validate subtle cranial and spinal abnormalities, distinguish true SIH-related dynamic changes from nonspecific variants, and reduce misclassification due to heterogeneous definitions of MRI-negative status.

## Conclusion

SIH presenting without typical brain MRI findings represents a challenging diagnostic scenario. Available data show that the cases with a negative MRI may show longer duration, higher recurrence, and sometimes more complex leak biology. Moreover, it is important to highlight that the absence of classical brain features does not exclude SIH and may support consideration of empiric treatment in carefully selected high-probability cases. Advanced imaging, including MR myelography, intrathecal gadolinium, and especially DSM, has expanded the diagnostic yield and clarified occult mechanisms such as CSF–venous fistulas, demonstrating the need for more investigations to reach a diagnosis of SIH in a subgroup of patients.

The therapeutic implications are relevant as well. EBP may be considered in carefully selected high-probability cases, particularly when symptoms are typical and alternative diagnoses have been excluded, while persistent or recurrent cases should prompt renewed efforts to identify an occult leak or CSF–venous fistula, as targeted repair of demonstrated CVFs or ventral dural tears may provide substantial clinical improvement. Overall, our review shows that MRI-negative SIH requires a comprehensive diagnostic strategy that includes standard and advanced modalities, combined with a treatment approach that balances empiric treatment with the search and definitive closure of leaks. This approach acknowledges both the limitations of imaging and the therapeutic potential of tailored intervention, potentially improving diagnostic accuracy and clinical outcomes in this challenging diagnostic scenario.

## Data Availability

Data that support this study are available in the text and tables of this article (and its Supplementary material). Additional data can be provided upon request.

## Footnotes

1
https://mcguinlu.shinyapps.io/robvis/

2
https://www.gradepro.org
