# Supplementary material for: Spontaneous intracranial hypotension with negative brain MRI findings: a systematic review of diagnostic strategies and clinical outcomes
Source: Front Neurol. 2026 Jul 9;17:1858043. doi: 10.3389/fneur.2026.1858043 (PMC13391874; doi:10.3389/fneur.2026.1858043)
Supplement: Supplementary file 1 [file Table_1.DOCX]

**Search Strategy**

**Filters: Human, English**

**Search was done on 3rd September 2025**

**Pubmed:** ("Spontaneous Intracranial Hypotension"[Mesh] OR "intracranial hypotension"[Title/Abstract] OR "cerebrospinal fluid leak"[Title/Abstract] OR "CSF leak"[Title/Abstract]) AND ("Magnetic Resonance Imaging"[Mesh] OR "MRI"[Title/Abstract] OR "Magnetic resonance"[Title/Abstract]) AND ("negative"[Title/Abstract] OR "normal"[Title/Abstract] OR "unremarkable"[Title/Abstract] OR "non-diagnostic"[Title/Abstract]) —> **215 Results**

**Scopus:** (TITLE-ABS-KEY("Spontaneous Intracranial Hypotension") OR TITLE-ABS-KEY("intracranial hypotension") OR TITLE-ABS-KEY("cerebrospinal fluid leak") OR TITLE-ABS-KEY("CSF leak")) AND (TITLE-ABS-KEY("Magnetic Resonance Imaging") OR TITLE-ABS-KEY("MRI") OR TITLE-ABS-KEY("Magnetic resonance")) AND (TITLE-ABS-KEY("negative") OR TITLE-ABS-KEY("normal") OR TITLE-ABS-KEY("unremarkable") OR TITLE-ABS-KEY("non-diagnostic")) —> **447 results**

**Web of science**: TS=("Spontaneous Intracranial Hypotension" OR "intracranial hypotension" OR "cerebrospinal fluid leak" OR "CSF leak") AND TS=("Magnetic Resonance Imaging" OR "MRI" OR "Magnetic resonance") AND TS=("negative" OR "normal" OR "unremarkable" OR "non-diagnostic") **—>224 Results**

**886 total results** > **485 after automatic deduplication** using RAYYAN > 31 **after title/abstract screening** > 15 **after full-text screening**

886 results

→ After Duplicates screening: 485 results

→ After Title&Abstract screening: 31 results

→ After Full text screening: 15 results

Included studies: 15
